# Supplementary material for: Hormesis Responses of Photosystem II in Arabidopsis thaliana under Water Deficit Stress
Source: Int J Mol Sci. 2023 May 31;24(11):9573. doi: 10.3390/ijms24119573 (PMC10254036; doi:10.3390/ijms24119573)
Supplement: Supplementary file 1 [file ijms-24-09573-s001.zip › ijms-2401973-supplementary.pdf]

Hormesis Responses of Photosystem II in *Arabidopsis thaliana* under Water Deficit Stress

Ilektra Sperdouli[1,2], Georgia Ouzounidou[3], and Michael Moustakas[1*]

Table S1. The measured chlorophyll fluorescence parameters with their definitions

Parameter	Definition	Calculation
Fv/Fm	Maximum efficiency of PSII photochemistry	$(Fm - Fo)/Fm$
Φ_{PSII}	Effective quantum yield of PSII photochemistry	$(Fm' - Fs)/Fm'$
Φ_{NPQ}	Quantum yield of regulated non-photochemical energy loss in PSII	$Fs/Fm' - Fs/Fm$
Φ_{NO}	Quantum yield of nonregulated energy loss in PSII	Fs/Fm
Fv'/Fm'	Efficiency of the open PSII reaction centers	$(Fm' - Fo')/Fm'$
ETR	Electron transport rate	$\Phi_{PSII} \times PAR \times c \times abs$, where PAR is the photosynthetically active radiation, c is 0.5, and abs is the total light absorption of the leaf taken as 0.84
qp	Photochemical quenching, representing the redox state of the plastoquinone pool and the fraction of open PSII reaction centers	$(Fm' - Fs)/(Fm' - Fo')$
EXCEE	Excess excitation energy at PSII	$(Fv/Fm - \Phi_{PSII})/Fv/Fm$
$1- qp$	Excitation pressure	$1 - [(Fm' - Fs)/(Fm' - Fo')]$
